# Effects of Topical Thymoquinone in an Experimental Dry Eye Model

**DOI:** 10.4274/tjo.50146

**Published:** 2018-12-27

**Authors:** Tolga Kocatürk, Erol Erkan, İbrahim Meteoğlu, Mehmet Ekici, Aslıhan Karul Büyüköztürk, İrfan Yavaşoğlu, Harun Çakmak, Volkan Dayanır, Muharrem Balkaya

**Affiliations:** 1 Adnan Menderes University Faculty of Medicine, Department of Ophthalmology, Aydın, Turkey; 2 Yozgat City Hospital, Ophthalmology Clinic, Yozgat, Turkey; 3 Adnan Menderes University Faculty of Medicine, Department of Pathology, Aydın, Turkey; 4 Adnan Menderes University Faculty of Veterinary Medicine, Department of Basic Sciences of Veterinary Medicine, Division of Veterinary Physiology, Aydın, Turkey; 5 Adnan Menderes University Faculty of Medicine, Department of Biochemistry, Aydın, Turkey; 6 Adnan Menderes University Faculty of Medicine, Department of Hematology, Aydın, Turkey; 7 Batıgöz Hospital, Ophthalmology Clinic, İzmir, Turkey

**Keywords:** Black seed oil, dry eye, thymoquinone

## Abstract

**Objectives:**

To comparatively evaluate the effects of thymoquinone (TQ), the biologically active main component of volatile oil derived from *Nigella sativa* seeds, in an experimental dry eye model.

**Materials and Methods:**

A total of 36 BALB/c mice 10 weeks of age were used in the study. The mice were divided into 6 groups of 6 mice. Two groups were negative and positive controls, and the other 4 groups were treated with balanced salt solution, fluorometholone (FML), TQ, or vehicle (Tween80). After 1 week of treatment, the mice were killed and the eyes removed for histopathologic examination and cytokine analysis. Interleukin (IL)-1α tumor necrosis factor-α, interferon-γ, IL-2, IL-6, IL-10, and lactoferrin levels in the conjunctival tissue were measured by multiplex immunobead assay. The presence of inflammatory cells in ocular tissue samples were investigated by hematoxylin-eosin and periodic acid-Schiff staining. Inflammatory T cells containing CXT receptor in the conjunctiva were determined by flow cytometry.

**Results:**

FLML and TQ groups had less inflammatory cell density and more goblet cells compared to the other groups. High levels of IL-1α and IL-2 were found in the TQ group.

**Conclusion:**

TQ treatment was associated with reduced inflammation in pathological examination, but did not significant lower cytokine levels.

## Introduction

Dry eye is a chronic and common eye problem that affects millions of people due to various reasons.^1^ Various factors including age, hormone deficiencies, medications, surgery, and systemic autoimmune diseases cause damage to lacrimal functional units and create surface inflammation, which can lead to dry eye symptoms.^2^

Chronic immune-mediated inflammatory processes in the lacrimal gland and conjunctival epithelium gradually cause dysfunction leading to destruction and are involved in the pathogenesis of dry eye. Increased levels of inflammatory cytokines, particularly interleukin 6 (IL-6), have been found in the lacrimal gland and conjunctival epithelium and/or in the patient’s tear fluid.^3,4,5,6,7^ Surface inflammation occurs due to functional lacrimal unit dysfunction and alterations in tear film composition and stability, leading to dry eye disease. Decreased tear production and removal creates a chronic ongoing inflammation cycle.^3^

There is currently no ideal treatment for dry eye disease. Artificial tears containing preservatives or tear support are the main treatment options, but they are palliative measures. Anti-inflammatory treatments for dry eye target mediators or pathways associated with dry eye.^8,9^ The use of topical preservative-free corticosteroids or cyclosporine may alleviate dry eye symptoms and provide normalization of the ocular surface.^10,11,12,13,14,15,16^ However, current treatment options are limited due to undesired side effects and low effectiveness. Possible side effects of immunosuppressive agents have not been fully established.

Thymoquinone (TQ) is the biologically active main constituent of volatile oil derived from black seed (*Nigella sativa*, also known as black cumin, fennel flower, and various other names), which is popular in Middle Eastern countries. It possesses strong antioxidant properties against oxidative damage induced by a variety of free radical-generating agents. The anti-inflammatory,^17,18^ antioxidant,^19,20^ and antineoplastic^21^ effects of TQ have been demonstrated both *in vivo* and *in vitro.*^22^ TQ appears to inhibit PGE_2 _production in arachidonic acid metabolism catalyzed by COX-2 more strongly than indomethacin.^18,23^

TQ is proposed to disrupt the pathogenetic mechanisms of dry eye disease through its anti-inflammatory effects. It may also theoretically have a lower side effect profile compared to other agents used in dry eye disease, such as steroids. There is no information available about the therapeutic potential of TQ in ocular tissues.

This research is a pioneer study on TQ and dry eye treatment. The study aimed to comparatively investigate the possible therapeutic efficacy and side effects of black seed oil, which naturally contains high levels of TQ and is easily accessible by the public, in dry eye disease.

## Materials and Methods

This study was approved by Institutional Animal Ethics Committee. The project was supported by the Scientific and Technological Research Council of Turkey (Türkiye Bilimsel ve Teknolojik Araştırma Kurumu [TÜBİTAK]; program code: 1002, project number: 214S539). A total of 36 male BALB/c mice approximately 10 weeks of age were used. The mice were randomly assigned to 6 equal groups. One group was a normal control group, while the experimental dry eye (EDE) model was applied in the other 5 groups. The mice in the 6 groups were subjected to the following EDE/treatment conditions: Group 1 (negative control group): no EDE + no treatment; Group 2 (positive control group): EDE + no treatment; Group 3: EDE + sterile Balanced Salt Solution (BSS) (Alcon, Fort Worth, TX, USA); Group 4: EDE + topical 0.1% fluorometholone (FML) (FML^®^, Allergan, Westport, County Mayo, Ireland); Group 5: EDE + TQ^8^; Group 6 (vehicle group): EDE + topical 0.8% Tween80 (vehicle). Following induction of EDE, approximately 2 µL eye drops were instilled twice daily for 1 week. After this treatment period, the mice were assessed by Schirmer test and tear break-up time (TBUT) assessment to measure tear production and stability, respectively. The mice were then killed; from each animal, one eye was prepared for flow cytometry and multiplex immunobead assay applications and the other for histopathological examination.

### Experimental Dry Eye Induction

EDE was induced in the mice by administering 0.2% benzalkonium chloride (BAC) topically to the eyes for 7 days.^24,25^

### Preparation of TQ

TQ powder (274666-1G, 2-isopropyl-5-methylbenzoquinone the Sigma-Aldrich, St. Louis, MO) was dissolved in 0.8% Tween80 at a dose of 0.4% and applied topically twice a day to the eyes of mice.^8^ Erdurmuş et al.^8^ reported that 0.4% TQ was as effective as triamcinolone acetonide.

### Tear Volume Measurement

Tear measurement was performed using a modified Schirmer I test. The lower eyelid was pulled down and Whatman 41 filter paper (trimmed by about ¼ to fit mouse eyes) was placed in the palpebral conjunctiva of the lower fornix (at the junction between the middle and temporal thirds). Distance wetted (mm) was measured at 15-second intervals while the eyes remained open. The measurement was repeated 3 times and the average was recorded.

### Tear Break-up Time Measurement

TBUT was measured as the time (s) from the first blink after instilling 1 µL sodium fluorescein in the lower conjunctival fornix to the appearance of the first dry spot on the cornea. Measurements were repeated three times and mean values were recorded.

### Tissue Collection

The mice were killed under ketamine (50 mg/kg) and xylazine (10 mg/kg) anesthesia. The right eyes were removed and fixed in 10% neutral buffered formalin (NBF) for histopathologic examination. The left eyes were obtained for multiplex immune bead assay and flow cytometry; they were homogenized and used for cytokine analysis.

### Histopathological Studies

Bulbar conjunctiva, palpebral conjunctiva, and lacrimal gland samples were examined histologically for inflammatory cells and goblet cells using hematoxylin and eosin stain and periodic acid-Schiff slides. The samples were evaluated in terms of presence of inflammatory cells and goblet cell density in the upper and the lower conjunctival regions.

### Enzyme-linked Immunosorbent Assay

The bulbar and the palpebral conjunctival tissue samples were lysed by incubating in radioimmunoprecipitation assay lysis buffer (50 mM Tris-HCl pH 7.5, 150 mM NaCl, 1% NP-40, and 0.1% sodium deoxycholate 1 µg/mL aprotinin, 1 µg/mL leupeptin) for 30 minutes. After centrifugation the obtained cell extracts and supernatants were stored at -80 °C until analysis. Related pro-inflammatory cytokine levels in the samples were assessed by ELISA using commercial kits (E-Bioscience, Platinium ELISA, Vienna, Austria) as per the manufacturers’ recommended protocol.

The supernatants were assessed for levels of IL-1α, tumor necrosis factor-alpha (TNF-α), IL-6, IL-2, lactoferrin, IL-10, and interferon-gamma (INF-γ). The basic 3-step working principle of the assay is as follows: An anti-mouse cytokine antibody was adsorbed onto microwells. Mouse cytokine present in the sample bound to the antibodies coating the microwell. A biotin-conjugated anti-mouse cytokine antibody was added to bind to the mouse cytokine captured by the first antibody (first incubation). Following incubation, unbound biotin-conjugated anti-mouse cytokine antibody was removed during a wash step. Streptavidin horseradish peroxidase (HRP) was added to bind to the biotin-conjugated anti-mouse cytokine antibody (second incubation). Following this incubation, unbound Streptavidin HRP was removed with a wash step, and a substrate solution reactive with HRP was added to the wells (third incubation). A colored product was formed in proportion to the amount of mouse cytokine present in the sample. The reaction was terminated by addition of acid and absorbance was measured at 450 nm. A standard curve was prepared from 7 mouse cytokine standard dilutions and the concentration of mouse cytokine in the sample was determined.

### Flow Cytometry

The amount of CD4+ inflammatory T cells containing CXC receptor 3 ligands in the bulbar and palpebral conjunctiva were determined by flow cytometry. In these cells, CD4, CD8, CD45, CD25, CD16, CD56, and CD3 were evaluated by flow cytometry using commercial kits (FITC, LS-C140363).

### Statistical Analysis

The data were analyzed using ANOVA. Tukey’s test was used for *post hoc* analysis.

## Results

**Figure 1:**
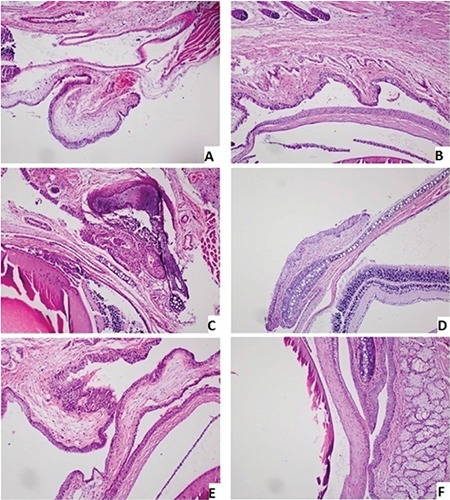
Histopathological findings (x100, H&E): A) Control: A few inflammatory cells and edema; B) EDE Control: Focal mild chronic inflammation; C) EDE+BSS: Intense chronic inflammation; D) EDE+FML: Focal mild chronic inflammation; E) EDE+TQ: Focal mild inflammation; F) EDE+Tween80: Slight to moderate inflammation EDE: Experimental dry eye, BSS: Balanced salt solution, TQ: Thymoquinone, FML: Fluorometholone, SD: Standard deviation

The data obtained are summarized in Tables 1 and 2. Figure 1 shows examples of the microscopic findings in each group.

### Tear Production

Table 1 summarizes the average tear production before and after treatment. There was a statistically significant difference between the results of Schirmer tests performed before and after the treatment (p<0.001). Repeated measures ANOVA confirmed the effect of time for the right eye (p=0.019), but the effect of time in the left eye was not statistically significant (p=0.084, f=3.190). In addition, the interaction between time and interventions was significant for both eyes (p<0.001). Within-subject test confirmed the effect of time and its interaction with interventions for the right eye (p=0.019 and p<0.001, respectively), but not for the left eye (p=0.084, f=3.190). *Post hoc* tests revealed that the differences were usually due to the control group. The average tear production of the negative control group was significantly greater than that of all other groups.

### Pathology

Tissue samples taken from the upper and lower conjunctiva and lacrimal gland were stained with hematoxylin and eosin and periodic acid-Schiff and evaluated in terms of inflammatory cell density and goblet cell numbers. The EDE control group had more inflammatory cells and fewer goblet cells. Among the treatment groups, the FML and TQ groups had lower inflammatory cell density and more goblet cells compared to the other groups. However, the differences were not significant.

### Pro-inflammatory Cytokines

Conjunctival IL-1α, TNF-α, IFN-γ, IL-2, IL-6, IL-10, and lactoferrin levels are summarized in Table 2.

Statistical analysis of the data indicated that interventions had a significant impact on IL-1α and IL-2 levels (p<0.001). However, their effect on IL-10 level was not statistically confirmed (p=0.065, f=2.353). *Post hoc* analysis showed that differences usually arose from the EDE+Tween80 and EDE+TQ groups. Especially in the EDE+TQ group, IL-1α levels were significantly higher compared to the other groups. The average IL-1α levels of the EDE+TQ group were higher when compared to the negative control, EDE control, EDE+BSS, and EDE+FML groups (p<0.001, p<0.001, p=0.026, and p=0.001, respectively). Similarly, the EDE+Tween80 group had higher mean IL-1α levels than the negative control, EDE control, and EDE+FML groups (p<0.001, p=0.001, and p=0.003, respectively). However, compared with EDE+BSS group, the increase in mean IL-1α levels of EDE+Tween80 group was not confirmed statistically (p=0.058, 95% CI: -742.4975-71973.7275). 

The difference in IL-2 levels also originated from the EDE+Tween80 and EDE+TQ groups. Tween80 administration caused an important increase in IL-2 levels compared to the control group and FML group (p=0.005 and p=0.046, respectively). However, the difference in mean IL-2 level between the BSS and Tween80 groups was not statistically significant (p=0.055; 95% CI: -27.049-3637.399). There was no significant difference in mean IL-2 levels between the EDE+Tween80 and control groups (p>0.05). However, similar to that of IL-1α, TQ application resulted in a significant increase in mean IL-2 levels compared to the negative control, EDE control, EDE+BSS, and EDE+FML groups (p=0.001, p=0.044, p=0.008, and p=0.006, respectively).

IL-6, INF-γ, and lactoferrin levels were also affected by the intervention, but nonsignificantly (p>0.05). When compared to the control group, Tween80 application also caused an increase in IL-6 values, but it was not statistically significant (p=0.063, 95% CI: -31.626-1,793.493).

Because flow cytometry values for most of the animals in each group were “out of range”, the data could not be evaluated statistically.

## Discussion

Dry eye disease is a common health problem in the vast majority of society. It occurs in older adults and in autoimmune disorders including Sjögren’s syndrome, and can also be seen after keratoplasty and chemotherapy. There are no curative treatment options besides temporary and palliative efforts. Therefore, the search for a permanent and continuous treatment option is ongoing. We investigated the possible therapeutic effects of TQ in an EDE model.

There are alternative measurement techniques to evaluate tear film volume, such as fluorescein staining, rose Bengal staining, phenol red thread test, Schirmer test, tear meniscus height, and TBUT.^26^ Clinically, no single test is capable of reliably differentiating individuals with and without dry eye. The Schirmer test is the most common test used in clinical practice to measure the quantity of aqueous tear production. Besides clinical workup, it is also used for experimental studies involving humans and animals.

Studies evaluating the effects of topical *Buddleja officinalis *extract on tear production in a castration-induced dry eye model in male Wistar rats^20^ and Victory rabbits^27^ revealed a significant increase in Schirmer values compared to untreated controls. Moreover, *B. officinalis *extract instillation decreased TNF-α expression in the lacrimal glands, while there was a significant increase in TGF-1β expression. In other words, *B. officinalis *extract also possesses anti-inflammatory effects.^27^

Corneal bioavailability may be another issue regarding the unexpectedly low effect of TQ on inflammatory cytokines. Topical administration in the eye is the most common and acceptable treatment route for various ocular diseases. However, the major problem of ophthalmic drug delivery is rapid elimination from the pre-ocular area due to anatomical constraints, such as lacrimal secretion, nasolacrimal drainage, and poor corneal permeability. Consequently, only a small amount of drug (1%-10%) permeates through the cornea into the intraocular tissues.^28^ TQ is known to be a lipophilic substance. In order to achieve rapid and adequate penetration, a drug must have an optimal lipophilic/hydrophilic balance in molecular structure.^28^ We used Tween80 as a solvent to enhance the corneal penetration of TQ.

Among the most popular drug delivery systems for ophthalmic application, lipid nanoparticles have recently received substantial attention. Solid lipid nanoparticles (SLN) and nanostructured lipid carriers (NLC), regarded as the first and second generation of lipid nanoparticles, respectively, have emerged as promising approaches to deliver drugs due to their ability to prolong the residence time of dosage forms, reduce systemic absorption and administration frequency, and enhance the bioavailability of drugs.^28^ Instead of Tween80, SLNs or NLCs might have increased the efficacy of TQ by enhancing its bioavailability.

One study showed that TQ had an inhibitory effect comparable to corticosteroids in a rat model of corneal neovascularization.^8^ This inhibition was dose-dependent and its mechanism, although not exactly known, may arise through antioxidant, anti-inflammatory, and immune regulatory factors.

In this study, Tween80 was used to dissolve powdered TQ to facilitate its passage through the corneal tissue. We prepared the TQ-Tween80 suspension as described previously by Erdurmuş et al.^8^ Different environmental factors, such as contamination with other chemicals, humidity, and temperature, may alter the concentration and purity of the TQ-Tween80 suspension. Impurity of the suspension may have resulted in unexpectedly low anti-inflammatory effects of TQ. Moreover, this impurity may have caused irritation on the ocular surface and altered the levels of inflammatory and anti-inflammatory cytokine levels as well. We used a concentration of 0.4%, the same as Erdurmuş et al.^8^ used for the treatment of corneal neovascularization. The dosage used in our study might have been excessive for the treatment of DED and resulted in adverse toxic and inflammatory effects.

Different animal models^29^ have been developed to imitate the pathophysiologic mechanisms involved in dry eye. Each has unique features and limitations, but none exactly matches the pathogenesis of human dry eye. BAC is a well-known preservative commonly used in eye drops and has long been recognized as a potential risk factor for dry eye syndrome because it causes serious ocular surface inflammation. As the major role of inflammation in dry eye is well known, the BAC-induced mouse model seems to be especially appropriate for studies evaluating the therapeutic effects of anti-inflammatory agents on ocular surface inflammation. We preferred to use the 0.2% BAC-induced dry eye model described by Xiong et al.^25^

Onizuka et al.^30^ conducted a study to examine the ophthalmic additives responsible for modulating acute corneal epithelial toxicity induced by BAC.^30^ Rabbit corneal epithelial cells were examined using the cell proliferation assay. Corneal damage was assessed using scanning electron microscopy. Among the tested additives, only Tween80 prevented BAC-induced cytotoxicity. Corneal epithelial barrier function disorder caused by 0.02% BAC was significantly alleviated by Tween80. In our study, only the group treated with Tween80 showed better results than the TQ group. Excessive TQ concentration or contamination from the environment might have caused the unexpected results in the TQ group. However, we found low levels of inflammatory cytokines, which supported Onizuka et al.’s^30^ results.

Despite the positive effect of Tween80 on corneal epithelial cells mentioned above, there are also some reports of anaphylactoid reactions caused by Tween80. Although a drug-induced anaphylactoid reaction is difficult to assay *in vitro *and in conventional animal models, Yang et al.^31^ developed a microplate-based quantitative *in vivo* zebra fish assay for assessing anaphylactoid reaction, and quantitatively measured live whole zebra fish mast cell tryptase activity. They concluded that impurity due to oxidized fatty acid residues in Tween80 samples, but not Tween80 itself, may induce anaphylactoid reaction.^31^

IL-6 is a mediator in inflammatory and allergic pathways. When challenged with conjunctival provocation with airborne allergens, atopic keratoconjunctivitis patients showed significant increases in IFN-γ, IL-6, and IL-10 in the tear fluid at 48 hours after provocation.^32^ Our results also showed marked elevation in IL-6 levels in the Tween80 group compared to the other groups. However, Tween80 application did not cause a statistically significant increase in IL-6 values when compared to the control group (p=0.063, 95% CI: -31.6266-1,793.4933).

Based on the well-known anti-inflammatory^17,18^ and antioxidant^19,20^ effects of TQ, positive effects on local inflammation and tear secretion were expected. The effects of TQ on dry eye and related inflammatory conditions were evaluated with biochemical and histopathological methods.

According to our data, artificial tear interventions had a partial positive effect when tear production was taken into account. In pathological examination, a reduction in inflammation was observed with TQ treatment; however, when compared to controls for the variables in the cascade of inflammation, high levels of IL-1α and IL-2 were seen in the TQ group, which may indicate systemic inflammation.^33^ One of the reasons for these findings may be inappropriateness of the substance for use on the ocular surface. When carefully examined, similar findings can be seen with Tween80. The TQ or Tween80 used in this study may not be pure enough to use in the eye; pyrogens in the solution may have triggered inflammation, thus limiting improvement in tear secretion caused by TQ. Another reason may be that TQ was administered at a lower dose than needed. Topical application of TQ helped reduce inflammation pathologically, but might not have been enough to reduce systemic inflammation. This study can serve as a basis for further studies examining these relationships.

## Conclusion

The method used in this study effectively induced EDE in BALB/c mice. Our pathological findings confirmed that groups treated with FML and TQ had lower inflammatory cell density, suggesting an anti-inflammatory effect of TQ on the eye. However, TQ was not associated with significant reduction in cytokine levels. 

## Figures and Tables

**Table 1:**
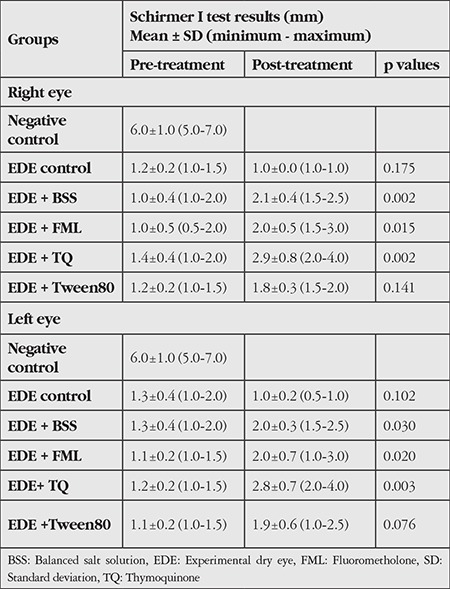
Mean tear production before and after treatment measured by Schirmer I test

**Table 2:**
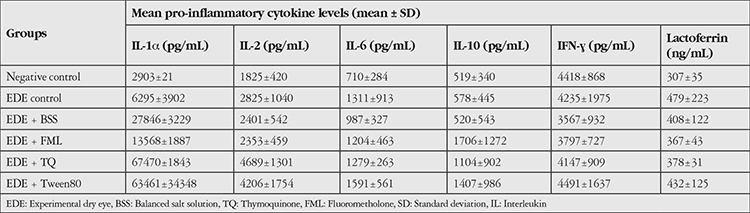
Mean levels of proinflammatory cytokines
